# Localization of neonatal Fc receptor for IgG in aggregated lymphoid nodules area in abomasum of Bactrian camels (*Camelus bactrianus*) of different ages

**DOI:** 10.1186/s12917-016-0847-9

**Published:** 2016-10-20

**Authors:** Wang-Dong Zhang, Wen-Hui Wang, Shu-Xian Li, Shuai Jia, Xue-Feng Zhang, Ting-Ting Cao

**Affiliations:** College of Veterinary Medicine, Gansu Agricultural University, Lanzhou, Gansu 730070 China

**Keywords:** Neonatal Fc receptor (FcRn), Expression, Aggregated lymphoid nodules area (ALNA), Bactrian camels, Epithelium, Mucosal immunity

## Abstract

**Background:**

The neonatal Fc receptor (FcRn) plays a crucial role in transporting IgG and associated antigens across polarized epithelial barriers in mucosal immunity. However, it was not clear that FcRn expression in aggregated lymphoid nodules area (ALNA) in abomasum, a unique and important mucosal immune structure discovered only in Bactrian camels. In the present study, 27 Alashan Bactrian camels were divided into the following five age groups: fetus (10–13 months of gestation), young (1–2 years), pubertal (3–5 years), middle-aged (6–16 years) and old (17–20 years). The FcRn expressions were observed and analyzed in detail with histology, immunohistochemistry, micro-image analysis and statistical methods.

**Results:**

The results showed that the FcRn was expressed in mucosal epithelial cells of ALNA from the fetus to the old group, although the expression level rapidly declined in old group; moreover, after the ALNA maturated, the FcRn expression level in the non-follicle-associated epithelium (non-FAE) was significantly higher than that in FAE (*P* < 0.05). In addition, the FcRn was also expressed in the vessel endothelium, smooth muscle tissue, and macrophages and dendritic cells (DCs) of secondary lymphoid follicles (sLFs).

**Conclusions:**

It was demonstrated that FcRn was mainly expressed in non-FAE, the effector sites, although which was expressed in FAE, the inductive sites for mucosal immunity. And it was also expressed in DCs and macrophages in sLFs of all ages of Bactrian camels. The results provided a powerful evidence that IgG (including HCAb) could participate in mucosal immune response and tolerance in ALNA of Bactrian camels through FcRn transmembrane transport.

## Background

In mucosal immunity, polymeric immunoglobulin receptor (pIgR) is an important receptor, which helps to maintain mucosal barrier integrity and gastroenteric homeostasis by transporting secretory immunoglobulin A (SIgA) antibodies across intestinal epithelial cells (IECs) into gut secretions [1–5]. As well, the neonatal Fc receptor (FcRn) also plays a crucial role in transporting IgG and associated antigens across polarized barriers [6–11]. It is another important receptor regulating mucosal immune response.

FcRn, originally discovered in the intestinal epithelium of newborn rat [12], is also referred to as the major histocompatibility complex (MHC) class I-related receptor due to their structural similarities [13]. The heterodimer composed of a soluble light chain β2-microglobulin (β2m) and a membrane-bound heavy chain that consists of three soluble domains (α1, α2 and α3), a single transmembrane helix, and a small cytoplasmic domain [14]. Although FcRn was originally named according to its expression pattern in rodent IECs where it was first identified, FcRn is now known to be expressed throughout life in many different cell types across the body [15–25]. However, the FcRn expression characteristics markedly differ with different species. For instance, in human intestinal epithelial cells, FcRn is expressed in both fetus and adult [15, 26]. By contrast, it is only highly in newborns and the level rapidly declines after weaning in mouse [27, 28]. In addition, the range of animals in which FcRn orthologs have also been identified includes rabbit [29], pig [30], sheep [31], bovine [32], Egyptian water buffalo [33], and dromedary [34].

Bactrian camel is an important livestock of economic characteristics in northwest of China and has some special immunological features. First, compared with the structure of conventional IgG, the Camelidae IgG2 and IgG3 are special heavy chain antibodies (HCAbs) [35], which are naturally devoid of light chain and their antigen binding site only consists of a single domain [36]. Second, compared with other livestock [37], Chinese Alashan Bactrian camels have a unique aggregated lymphoid nodule area (ALNA) in the abomasum [38]. This species-specific anatomical structure could be divided into the reticular mucosal folds region (RMFR) and longitudinal mucosal folds region (LMFR) [38–40]. They belonged to the organized mucosa-associated lymphoid tissue (MALT). However, the FcRn expression in this region has not been reported at present. Based on our previous researches on the morphology and histology of ALNA [38–40], the characteristics of FcRn expression with age in this area were studied in this paper. We hope that it will provide the necessary immunomorphology support for further studying whether FcRn could participate in mucosal immunity in this area or not.

## Methods

### Experimental animals

Twenty-seven Alashan Bactrian camels of different ages (half male and half female, except fetus group) were divided into five age groups: fetus (10–13 months’ gestation, *n* = 3, two males and one female), young (1–2 years, *n* = 6), pubertal (3–5 years, *n* = 6), middle-aged (6–16 years, *n* = 6) and old (17–20 years, *n* = 6). Fetus tissues were collected from animal carcasses submitted to the necropsy service in College of Veterinary Medicine, Gansu Agricultural University. Other animals were from the slaughterhouse (Xining, Qinghai province of China) and were not starved before slaughter, which were anaesthetised intravenously with sodium pentobarbital (20 mg/kg) and then exsanguinated to death.

### Microsection

The whole abomasum from the isthmus to the pyloric ostium was incised along the greater curvature. The gastric contents were cleaned with saline. Samples from RMFR and LMFR of ANLNA were rapidly taken after death and fixed in 10 % neutral formalin. The paraffin sections were made and stained with haematoxylin and eosin (H&E) by a routine method [41] as well as SABC-immunohistochemistry (IHC) by the method as follows: the samples were sectioned (4 μm) and placed on the polylysine-coated slides (molecular weight: 150, 000–300,000; working concentration: 0.10 % (w/v) solution in water, Sigma, USA). After deparaffination, we used 1.0 mg/ml trypsin 1: 250 (250.N.F.U/mg, Sigma, USA) for enzyme-induced epitope retrieval, which was followed by endogenous peroxidase blocking (3 % H_2_O_2_). For blocking, 5 % bovine serum albumin (BSA, from easy-to-use immunohistochemical kit, Lot No.07H3OCJ, Boster, Wuhan, Hubei, China) was used. All samples with the primary antibody were incubated at 4 °C overnight. After being rinsed with PBS 2 min × 3 times; HRP conjugated goat anti-rabbit IgG (from easy-to-use immunohistochemical kit, Lot No.07H3OCJ, Boster, Wuhan, Hubei, China) as secondary antibody was applied for 1 h in humidified box at 37 °C. After being rinsed with PBS 5 min × 4 times. The SABC was applied for 30 min in humidified box at 37 °C. After being rinsed with PBS 5 min × 4 times. For detection, DAB Kit (ZSGB-BIO, Beijing, China) was used at room temperature. Slides were counterstained with Hematoxylin (Solarbio, Beijing, China) and mounted with Neutral Balsam (Solarbio, Beijing, China). Sections were examined with an Olympus microscope (Olympus, Hamburg, Germany) [42].

### Primary antibodies selection and analysis

Rabbit polyclonal antibodies against human FcRn, diluted with the buffer at 3.33 μg/ml before use, was supported by BIOSS (Lot No. 140226, BIOSS, Beijing, China).

Some studies reported that the drFcRn/ Fc contact residues were highly conserved, and the structures of FcRn in different species were similar [14, 34, 43]. Hence, the epitopes of FcRn are similar among different species. Moreover, in immune responses, MHCII presents antigens to CD4^+ ^T cells, and the antigen peptide should be composed of more than 12 amino acid residues. The molecular weight of human FcRn is about 50 kDa. Moreover, the similarity of FcRn between dromedary and human was 78.6 % by analyzing the genes of phylogenetic relatedness of the extracellular domains (α1–α2–α3) [34]. Therefore, this primary antibody well met the sequent experiment request.

### Second antibodies

SABC goat anti-rabbit IgG polyclonal antibodies immunohistochemical kit (Lot No.07H3OCJ, Boster, Wuhan, Hubei, China). The kit contained 1.5% BSA:12 ml. Second antibodies: Biotin goat anti rabbit IgG: 12 ml. SABC: 12 ml. It is an easy-to-use kit, which can be used directly and is unnecessary to be diluted with the buffer.

### Light microscopy

In each group, the expression sites and characteristics of FcRn were observed in detail under microscope and photomicrographed using Olympus DP-71 microscopy system.

### Statistical analysis

Five sections were randomly selected for each sample. Ten microscopic fields were randomly selected, observed and photomicrographed for FAE, non-FAE, vascular, smooth muscle and lymphoid follicle in each section. The mean optical density (MOD) of each site was calculated (Image-Pro Plus 6.0), respectively. The main steps contain: 1. the background correction of the IHC photos (this can make the light intensity in the central and around the IHC photos become consistent); 2. the correction of the optical density of the IHC photos (this can change the image intensity to the optical density); 3. the parameter setting (select the integrated optical density (IOD) as the measurement value of the ICH image); 4. selection the measurement region through the software tools; 5. color settings; 6. measurement of the IOD and the area of the selected region; 7. the calculation of the MOD through IOD/selected areas. The MOD differences among groups were analyzed by one-way ANOVA followed by Duncan’s new multiple range test using IBM SPSS v. 23.0 (SPSS Inc., Chicago, USA). The significant difference was considered at *P* < 0.05.

## Results

### Localization of FcRn in abomasum ALNA in Bactrian camels of different ages

#### Fetus group 

**Fig. 1:**
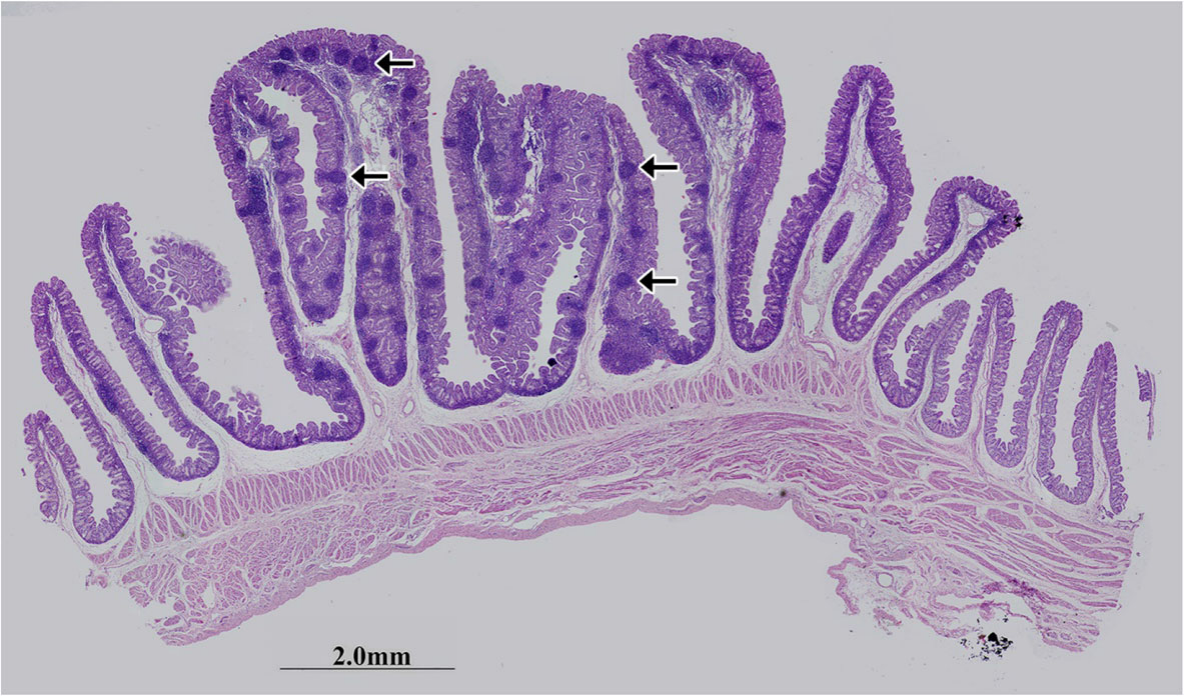
Histological characteristics of the RMFR in fetus group. A plenty of primary lymphatic follicles (pLFs) (*arrow*) were seen in this area and they were mainly distributed in the lamina propria (LP). Original magnification: 40×

**Fig. 2:**
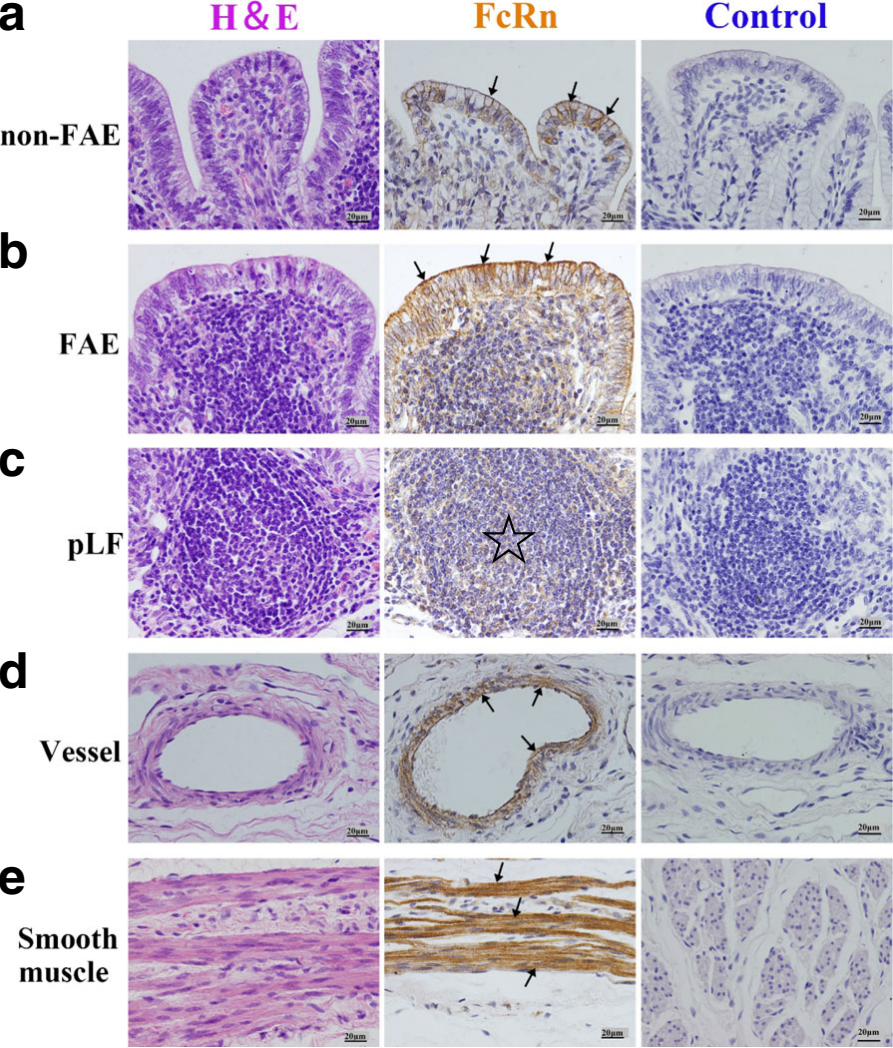
Localization of FcRn in RMFR of fetus’ abomasum ALNA. From left to right column, the paraffin sections were stained with hematoxylin and eosin (H&E), immunohistochemistry for FcRn, hematoxylin counterstain (as negative control), respectively. **a** non-FAE represents non-follicle-associated epithelium in the top panel, and FcRn positive expression was mainly distributed in membrane of the epithelial cells (*arrow*); **b** FAE represents follicle-associated epithelium in the second panel from top to bottom, and FcRn positive expression was mainly distributed in membrane of the epithelial cells (*arrow*); **c** pLF represents primary lymphatic follicles in the third panel (*star*), and typical macrophages and dendritic cells (DCs) with high FcRn expression were not observed; **d** Vessel, FcRn was highly expressed both in vascular endothelial cells and smooth muscle cells (*arrow*); **e** Smooth muscle, FcRn was also highly expressed in basement membrane of smooth muscle (*arrow*). Original magnification: 400×

**Fig. 3:**
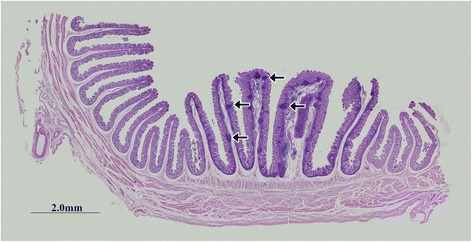
Histological characteristics of the LMFR in fetus group. A certain amount of primary lymphatic follicles (pLFs) (*arrow*) were seen in this area. The distribution characteristics of pLFs were similar to those in RMFR, i.e., they were mainly distributed in the lamina propria (LP). Original magnification: 40×

**Fig. 4:**
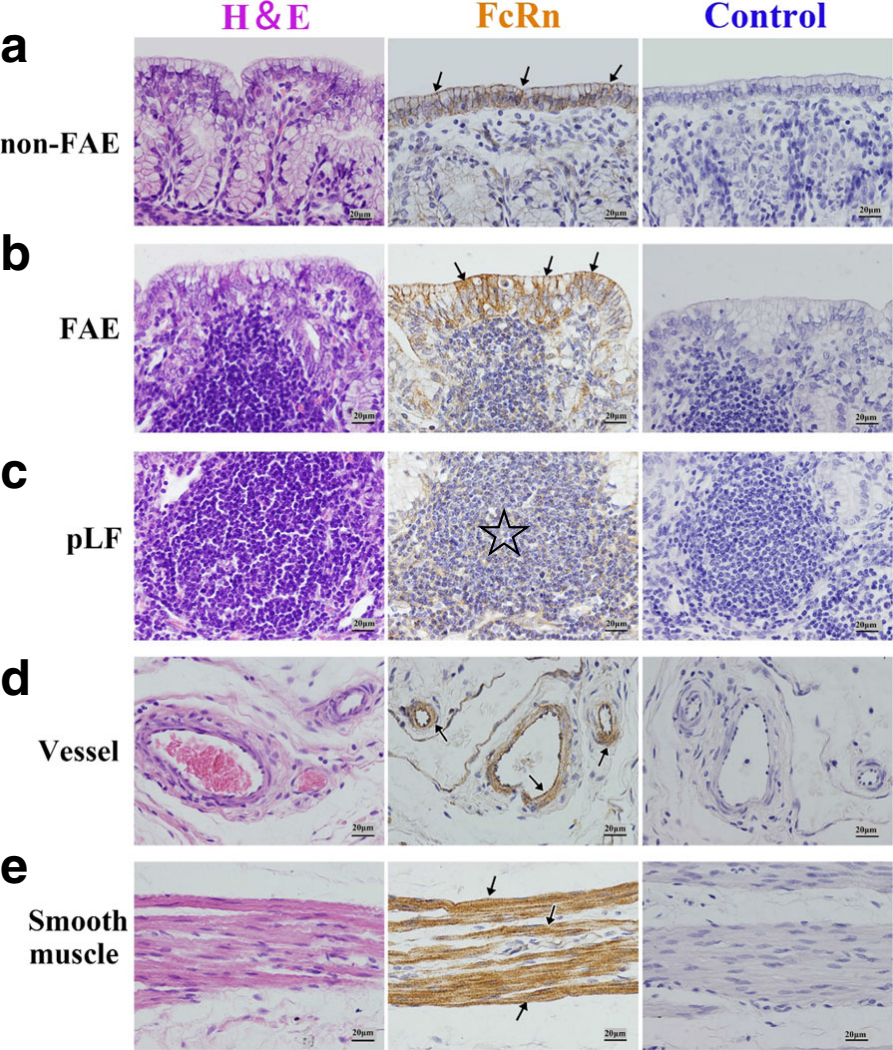
Localization of FcRn in LMFR of fetus’ abomasum ALNA. From left to right column, the paraffin sections were stained with hematoxylin and eosin (H&E), immunohistochemistry for FcRn, hematoxylin counterstain (as negative control), respectively. **a** non-FAE represents non-follicle-associated epithelium in the top panel, and FcRn positive expression was mainly distributed in membrane of the epithelial cells (*arrow*); **b** FAE represents follicle-associated epithelium in the second panel from top to bottom, and FcRn positive expression was mainly distributed in membrane of the epithelial cells (*arrow*); **c** pLF represents primary lymphatic follicles in the third panel (*star*), and typical macrophages and dendritic cells (DCs) with high FcRn expression were not observed; **d** Vessel, FcRn was highly expressed both in vascular endothelial cells and smooth muscle cells (*arrow*); **e** Smooth muscle, FcRn was also highly expressed in basement membrane of smooth muscle (*arrow*). Original magnification: 400×

﻿**(1')﻿**﻿ In RMFR, a plenty of primary lymphatic follicles (pLFs) were primary densely-distributed in the lamina propria (LP) (Fig. 1). And FcRn was expressed in both non-follicle-associated epithelium (non-FAE) (Fig. 2a) and FAE (Fig. 2b), and mainly at the apical membrane. However, typical macrophages and dendritic cells (DCs) with high FcRn expression were not observed in the pLFs (Fig. 2c). In addition, the FcRn was also expressed in vessel endothelium (Fig. 2d) and smooth muscle tissue (Fig. 2e). **(2')** In LMFR, the histological characteristics were similar to those in RMFR. Lymphatic follicles were also pLFs. But the distribution density was lower than that in RMFR (Fig. 3). The localization of FcRn was similar to that in RMFR (Fig. 4a–e).

#### Young group

**Fig. 5:**
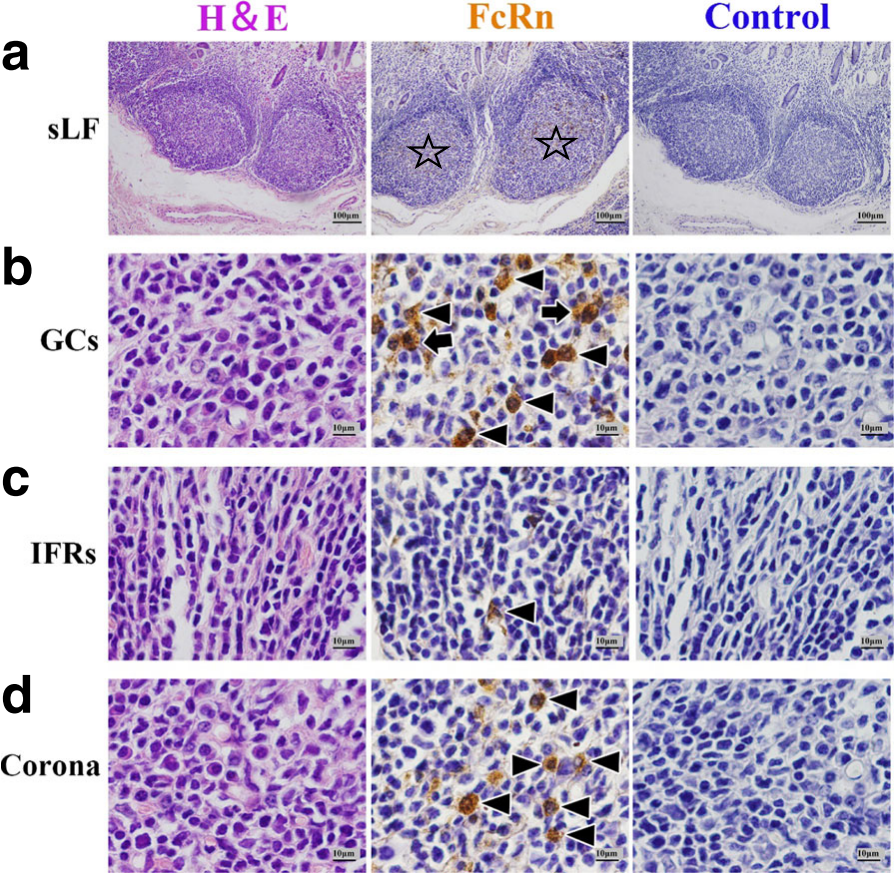
Macrophages and DCs highly expressed FcRn in sLFs in RMFR of young abomasum ALNA. From left to right column, the paraffin sections were stained with hematoxylin and eosin (H&E), immunohistochemistry for FcRn, hematoxylin counterstain (as negative control), respectively. **a** sLF represents second lymphatic follicles (*star*) (original magnification: 100×); **b** GCs represents germinal centers, and many macrophages (*arrow*) and dendritic cells (DCs) (*triangle*) with high FcRn expression were seen (original magnification: 1000×); **c** IFRs represents interfollicular regions, also called T-dependent area, and some DCs (triangle) with high FcRn expression were seen (original magnification: 1000×); **d** Corona represents corona area, also called B lymphocyte zone, and many DCs (*triangle*) with high FcRn expression were seen (original magnification: 1000×)

**Fig. 6:**
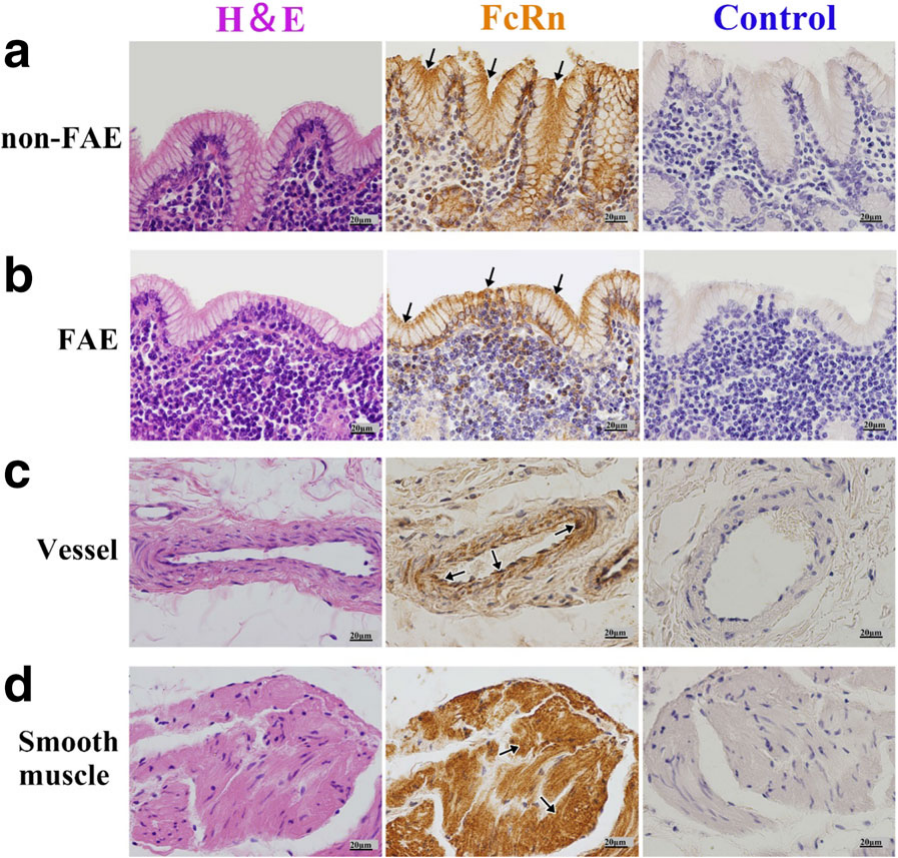
Localization of FcRn in RMFR of young abomasum ALNA. From left to right column, the paraffin sections were stained with hematoxylin and eosin (H&E), immunohistochemistry for FcRn, hematoxylin counterstain (as negative control), respectively. **a** non-FAE represents non-follicle-associated epithelium in the top panel, and FcRn positive expression was mainly distributed in membrane of the epithelial cells (*arrow*); **b** FAE represents follicle-associated epithelium in the second panel from top to bottom and FcRn positive expression was mainly distributed in membrane of the epithelial cells (*arrow*), but the expression level was lower than that in non-FAE; **c** Vessel, FcRn was highly expressed both in vascular endothelial cells and smooth muscle cells (*arrow*); **d** Smooth muscle, FcRn was also highly expressed in basement membrane of smooth muscle (*arrow*). Original magnification: 400×

**Fig. 7:**
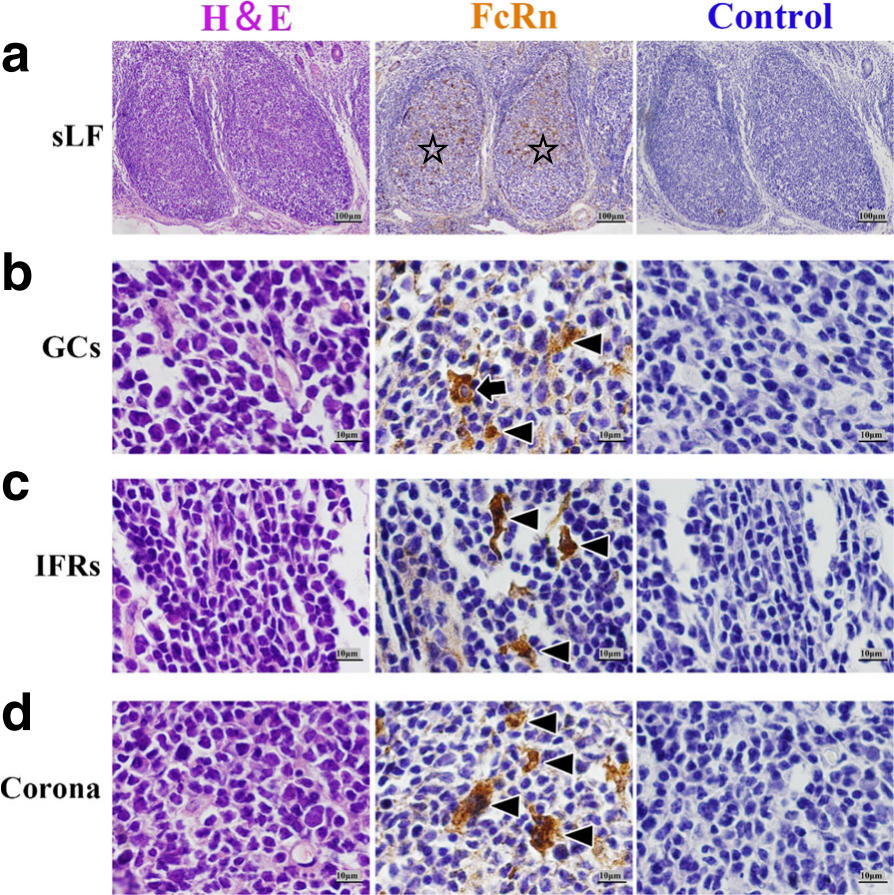
Macrophages and DCs highly expressed FcRn in sLFs in LMFR of young abomasum ALNA. From left to right column, the paraffin sections were stained with hematoxylin and eosin (H&E), immunohistochemistry for FcRn, hematoxylin counterstain (as negative control), respectively. **a** sLF represents second lymphatic follicles (*star*) (original magnification: 100×); **b** GCs represents germinal centers, and many macrophages (*arrow*) and dendritic cells (DCs) (*triangle*) with high FcRn expression were seen (original magnification: 1000×); **c** IFRs represents interfollicular regions, also called B lymphocyte zone, and some DCs (*triangle*) with high FcRn expression were seen (original magnification: 1000×); **d** Corona represents corona area, also called B lymphocyte zone, and many DCs (*triangle*) with high FcRn expression were seen (original magnification: 1000×)

**Fig. 8:**
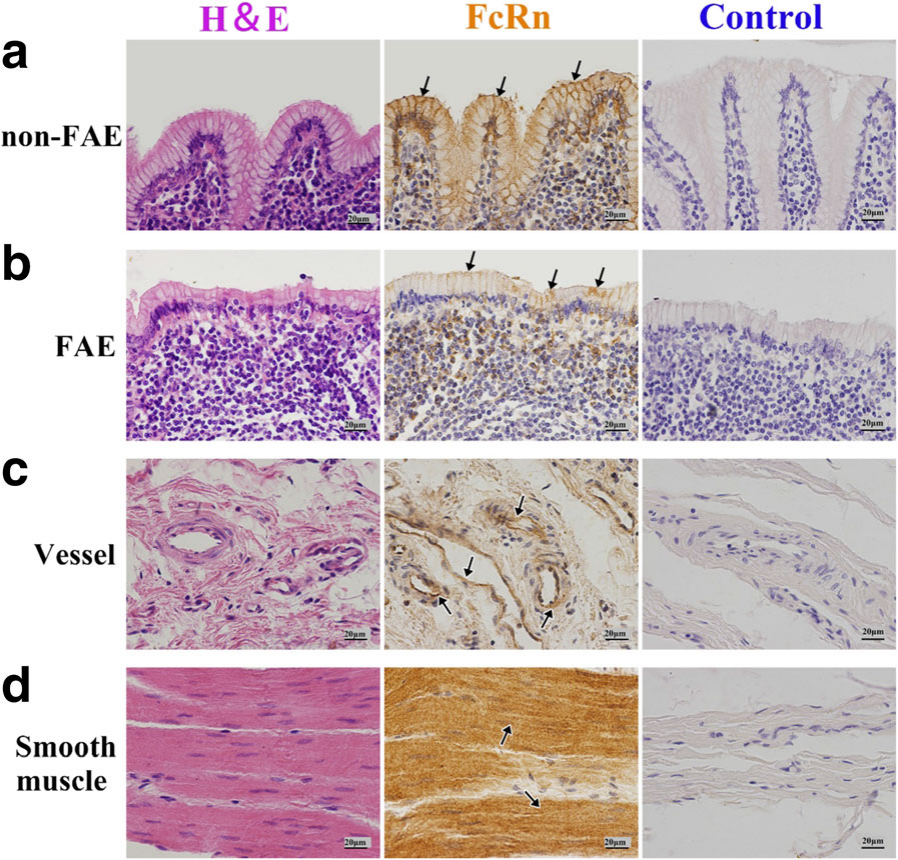
Localization of FcRn in LMFR of young abomasum ALNA. From left to right column, the paraffin sections were stained with hematoxylin and eosin (H&E), immunohistochemistry for FcRn, hematoxylin counterstain (as negative control), respectively. **a** non-FAE represents non-follicle-associated epithelium in the top panel, and FcRn positive expression was mainly distributed in membrane of the epithelial cells (*arrow*); **b** FAE represents follicle-associated epithelium in the second panel from top to bottom and FcRn positive expression was mainly distributed in membrane of the epithelial cells (*arrow*), but the expression level was lower than that in non-FAE; **c** Vessel, FcRn was highly expressed both in vascular endothelial cells and smooth muscle cells (*arrow*); **d** Smooth muscle, FcRn was also highly expressed in basement membrane of smooth muscle (*arrow*). Original magnification: 400×

**(1')** In RMFR, a plenty of secondary lymphatic follicles (sLFs) were concentrated in the LP and submucosa (Fig. 5a). FcRn was highly expressed in typical macrophages and DCs in sLFs (Fig. 5b, c and d). Meanwhile, the FcRn was also expressed in non-FAE (Fig. 6a), FAE (Fig. 6b), vessel endothelium (Fig. 6c) and smooth muscle tissue (Fig. 6d). **(2')** In LMFR, the localization of FcRn was similar to that in RMFR (Figs. 7 and 8).

#### Pubertal and middle-aged groups

The localization of FcRn was in the two groups were both similar to those in young group.

#### Old group

The localization of FcRn was in RMFR and LMFR were similar. It was highly expressed in macrophages and DCs in sLFs, however, the expression levels were very low in other tissues.

### FcRn expression levels in mucosal epithelium of abomasum ALNA with age

#### FcRn expression levels

**Fig. 9:**
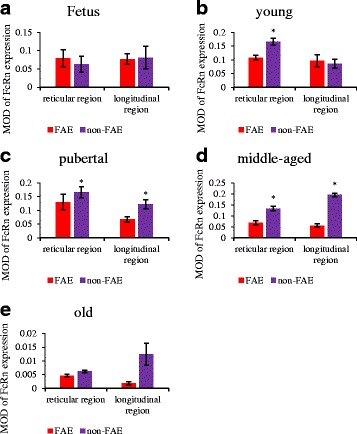
FcRn expression level in abomasum ALNA of Bactrian camels of different ages. Diagram **a** to **e** represent the FcRn expression levels in mucosa epithelium from fetus to old group, respectively. In RMFR, the expression levels of FcRn in non-FAE were significantly higher than those in FAE in young, pubertal and middle-aged groups, respectively (*P* < 0.05), but there were no significant differences in fetus and old groups (*P* > 0.05). In LMFR, the expression levels of FcRn in non-FAE were significantly higher than those in FAE in pubertal and middle-aged groups (*P* < 0.05), but there were no significant differences in fetus, young and old groups (*P* > 0.05). * *P* < 0.05, significant differences compared with FAE

The MOD value detection results showed: (**1')** In RMFR, the FcRn expression level in non-FAE was significantly higher than that in FAE in young, pubertal and middle-aged groups, respectively (*P* < 0.05) (Fig. 9b, c and d), but it had no significant difference in fetus and old groups (*P* > 0.05) (Fig. 9a and e); **(2')** In LMFR, the FcRn expression level in non-FAE was significantly higher than that in FAE in pubertal and middle-aged groups, respectively (*P* < 0.05) (Fig. 9c and d), and it had no significant difference in other groups (*P* > 0.05) (Fig. 9a, b and e).

#### Changes in the FcRn expression with age

**Fig. 10:**
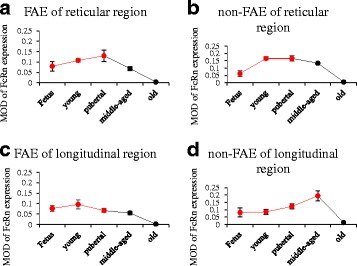
Changes in FcRn expression in mucosal epithelium of abomasum ALNA with age. Diagram **a** to **d** represent the changes in FcRn expression with age in FAE of RMFR, non-FAE of RMFR, FAE of LMFR, non-FAE of LMFR, respectively. Red lines represent increase with age, and black lines represent decrease with age. The results showed that in the FAE and non-FAE of RMFR, the FcRn expression level peaked at the pubertal period, and then significant decreased with age. The case was similar in the FAE of LMFR, however, the FcRn expression level in non-FAE of LMFR peaked at Middle-aged period, and then significant decreased with age. It probably related to the upregulation of compensatory reflection because of the FcRn expression level was decreased in other site in this period. In Old group, the FcRn expression level was very low, and it probably related to the degradation of the mucosal immune or immunosenescence

**﻿(1')** ﻿In RMFR, the FcRn expression level in FEA and non-FAE both gradually increased from fetus to pubertal groups with increasing age, peaked in pubertal group, and subsequently gradually declined (Fig. 10a and b). **(2')** In LMFR, the FcRn expression level in FAE peaked in young and kept the high level to pubertal period, then subsequently significantly declined, but that in non-FAE gradually increased with increasing age, peaked in the middle-aged group (Fig. 10c and d). In addition, the FcRn expression level significantly decreased in the mucosa epithelium in old group (*P* < 0.05) (Fig. 10e).

## Discussion

Organized MALT is a critical part of the mucosal immune system [44]. Wang et al. reported that there were developed ALNA in abomasum of Bactrian camel [38–40], which belonged to the organized MALT. Our result showed that FcRn was expressed in non-FAE of ALNA at different levels in all groups, which was compatible with the expression of FcRn in human enterocytes (FcRn was expressed in adult human enterocyte) [15]. However, this was very different from the FcRn expression in the intestinal mucosal epithelium of rat [8], which was only expressed in newborns enterocyte and rapidly declined after weaning in mouse. Researches have demonstrated that FcRn was pH-dependent binding to IgG, with relatively strong binding at acidic pH (pH ≤ 6.5) and negligible binding at physiological pH (7.3–7.4) [43]. Some studies reported that the pH was 5.55 in the camel abomasum [45], which was just within the range of optimal pH value for FcRn binding to IgG. Thus, FcRn expression in the mucosal epithelial cells of ALNA in abomasum of Bactrian camels of different ages could provide a powerful evidence for FcRn participating in the transmembrane transport of IgG and associated antigens (especially the transportation of HCAb).

Compared with non-FAE, the FAE is a kind of specialized epithelium, on which the unique microfolds cell (M cell) could efficiently uptake and transport macromolecules and microorganisms in gut lumen to the underlying lymphoid tissue [46]. The MOD measuring results of FcRn expression in epithelial cell of this area found that the FcRn expression level in FAE and non-FAE had no difference in the fetus and young groups, respectively (*P* > 0.05), while that in FAE was significantly lower than that in non-FAE in pubertal and middle-aged groups, respectively (*P* < 0.05). The expression characteristics were similar to those of pIgR, transport receptor of SIgA, in this area [46]. In ALNA of the fetus to middle-aged Bactrian camels, although the FcRn was expressed in mucosal immune inductive sites FAE, in ALNA of the fetus to old Bactrian camels, mainly in effector sites non-FAE. As for whether FcRn participated in M cells uptaking and transporting associated antigens in FAE of ALNA in abomasum of Bactrian camels remains a further study.

In addition, our results showed that FcRn was expressed in the vessel endothelium and smooth muscle in this area, which was similar to the FcRn expression characteristics in the vessel and smooth of mice and humans [47]. It suggested that the FcRn expression in these sites was mainly related to regulating the half-life of IgG and albumin and homeostasis in this local region.

In the present study, both macrophages and DCs in sLFs of ALNA in abomasum of Bactrian camels highly expressed FcRn, respectively. Researches have shown that there was mononuclear phagocyte system composed of monocytes, DCs and macrophages in sLFs. And in this system, different types of cells had different subtypes, respectively, and were distributed in special regions [48–53]. They played an important role in antigen capture, processing and presentation, secreting cytokines and regulating immune tolerance [54, 55]. These results provided an evidence that FcRn could participate in regulating the immune response and tolerance in the sLFs of ALNA in abomasum of Bactrian camels.

## Conclusions

Our results showed that FcRn was mainly expressed in non-FAE, the effector sites, although which was expressed in FAE, the inductive sites for mucosal immunity. And it was also expressed in DCs and macrophages in sLFs of all ages of Bactrian camels. This provided a powerful evidence that IgG could participate in mucosal immune and immune response and tolerance in this area.

## Abbreviations

FcRn: Neonatal Fc receptor
ALNA: Aggregated lymphoid nodules area
RMFR: Reticular mucosal folds region
LMFR: Longitudinal mucosal folds region
MALT: Mucosa-associated lymphoid tissue
MECs: Mucosal epithelial cells
non-FAE: Non-follicle-associated epithelium
DCs: Dendritic cells
pLFs: Primary lymphoid follicles
sLFs: Secondary lymphoid follicles
HCAb: Heavy chain antibody
IHC: Immunohistochemistry
MOD: Mean optical density
SED: Subepithelial dome
IFRs: Interfollicular regions
M cell: Microfolds cell
